# Meningitis Cerebro-Spinalis Subacuta

**Published:** 1889-08

**Authors:** 


### MENINGITIS CEREBRO-SPINALIS SUBACUTA.

In the Homœopathic Hospital at Leipzig, Dr. Stifft treated the following interesting case, which deserves our notice, as the disease is too often fatal.

Richard, twenty-four years old, complained Jan. 22d of stitches and heaviness of the legs, pain in sacrum, and nausea. He improved, and could again work at his trade of book-binder, when, February 11th, without cause, the same pain returned, with severe pains all along the spinal cord and in head, with excessive anguish and restless sleep; also singing in ears, disturbed vision, vomiting, loss of appetite, constipation. For two months the pains in the head were of such severity that patient screamed, vomiting after every meal, high fever, with excessively high temperature; stiffness and tearing pains in upper extremities. February 24th, stiffness in neck, with great pains when trying to sit up. Nightly deliria now set in, and in the beginning of March patient became more quiet, sleepy, with progressive emaciation. Admitted into the hospital, the patient showed a temperature of 38°, icy-cold extremities, perfect apathy, nearly coma; pressure on the spinal column, especially near the sacrum, very painful; breathing superficial, lips dry, pupils dilated. Belladonna 3d c., twelve drops in water.

March 4th.—Coma deeper. Zincum cyanat.$^3$, a small powder every three hours.

March 7th.—Patient sleeps a great deal, but can be aroused, skin warm, off and on, horripilations; vomiting of milk; stool after clysma; evening a high fever, with chills; temp. 38.7°; pulse 88°; breathing 30; screams "my head, my head." Continue.

March 8th.—Comatose in the morning, temp. 37.7°; vomiting; feels better toward evening; temp. 36.80°, pulse 60, breathing 16; no vomiting. Quiet sleep during the night.

March 9th.—Temp. normal, more appetite, complains less of head and back.

March 10th.—State the same; for the first time able to move the head; traces of albumen in the urine.

March 11th.—State the same, but vomiting; at noon temp. 39.5, pulse 88, respiration 26; more restlessness and more pain in head and back. Evening, temp. 38.7, pulse 88, respiration 26. More quiet toward morning.

March 12th.—Temp. normal, pulse 58.76, respiration 16–20; less pain; no vomiting, more quiet.

March 16th.—Steady improvement, able to converse and to move about in bed; hyperæsthesia less; wants meat; begins to feel his returning health. Discharged April 1st.

Gerstel, in the *International Homœopathic Presse*, iii and vii, shows that the pathogenesis of Zincum gives nearly all the principal symptoms of the case, and, by inductive reasoning, Stifft concluded: Zincum cyanatum, as well as Mercurius cyanatus, belong to those cyan-metals, which may be poisonous to the organismus, by setting Prussic acid free through the action of diluted Hydrochloric acid. A different state is found in Barium cyanat. and Ferrum cyanat., which are not poisonous. Just as Mercur. cyanat. shows its specific action in those cases of grave diphtheria, whereby the beginning of paralysis of the cerebro-spinal centres, and especially of the medulla oblongata, life is threatened, or perhaps only saved, by the action of the Hydrocyanic acid, which in large doses paralyzed these centres, but in minute doses increases their functions, and thus blood-pressure and the frequency of respiration, so also Cyanide of Zinc acts by being decomposed. That the case was grave, and that under old-school treatment most patients succumb, none will deny, and even when they recover the convalescence is usually tardy and protracted, while here the patient was able, after a few weeks, to return to his accustomed duties.—*Popular Zeitschr f. Hom., May,* '89.

